# Semantic Prior-Guided Period-Aware Multi-Expert Segmentation for Long-Term Fixed-View Visual Monitoring

**DOI:** 10.3390/s26175396

**Published:** 2026-08-26

**Authors:** Li Hao, Yanan Gan, Zeyu Jia, Shengling Geng

**Affiliations:** 1 School of Computer, Qinghai Normal University, Xining 810008, China; 202133341019@stu.qhnu.edu.cn (L.H.); 202133341018@stu.qhnu.edu.cn (Y.G.); 20191060@qhnu.edu.cn (Z.J.); 2 Academy of Plateau Science and Sustainability, People’s Government of Qinghai Province & Beijing Normal University, Xining 810008, China; 3 State Key Laboratory of Tibetan Intelligent, Qinghai Normal University, Xining 810008, China

**Keywords:** semantic segmentation, visual monitoring, fixed-view monitoring, period-aware segmentation, semantic priors, multi-expert learning, water-body segmentation

## Abstract

Accurate semantic segmentation is essential for long-term fixed-view monitoring, where seasonal, illumination, weather, and environmental changes alter local appearance while the global scene layout remains relatively stable. To address this structure–appearance modeling problem, we propose SPMES, a Semantic Prior-Guided Period-Aware Multi-Expert Segmentation framework. SPMES comprises a Global Expert that learns stable semantic-prior maps from the complete training set, a Semantic-Guided Fusion Module that injects these priors into the input representation, and period-specific experts that model recurring appearance characteristics according to acquisition time. Experiments on a long-term fixed-view monitoring dataset collected for this study evaluate SPMES with U-Net, U-Net++, U^2^-Net^†^, Swin-Unet, and Mamba-UNet. Compared with the corresponding baselines, SPMES obtains better point estimates across all six evaluation metrics for each backbone, although the magnitude of the changes varies across architectures and metrics. Ablation studies show that individual configurations exhibit metric- and backbone-dependent effects, whereas the complete integration of global semantic priors, semantic-guided fusion, and period-specific learning provides the best overall balance. These results support the effectiveness and backbone-level compatibility of SPMES within the studied monitoring setting.

## 1. Introduction

Vision-based monitoring systems are widely used for continuous observation, process analysis, and early-warning applications in environmental and engineering scenarios. In these systems, fixed-position cameras or visual sensors can continuously acquire images of the same target area over long periods. Semantic segmentation provides pixel-level scene understanding for such monitoring data and has been widely studied in image understanding, remote sensing analysis, medical image processing, and video surveillance [1,2,3,4,5]. Despite recent progress, segmentation in long-term fixed-view monitoring scenes remains challenging. Images acquired in different periods may show substantial appearance variations caused by seasonal changes, illumination differences, weather conditions, and environmental disturbances. These factors can change local texture, contrast, and boundary visibility, thereby weakening segmentation accuracy and structural stability [6,7,8].

A distinctive property of long-term fixed-view monitoring is that the camera viewpoint remains stable while the visual appearance of the monitored scene changes over time. Therefore, the global spatial layout and semantic structure of the monitored target are usually more stable than local appearance patterns. This property suggests that fixed-view segmentation should not only fit local appearance, but should also exploit stable scene-level semantic structure. However, a single model trained on all acquisition periods may average out period-specific appearance patterns, while independently trained period-specific models may suffer from limited samples and insufficient global structural knowledge. This creates a structure–appearance modeling problem: the model should preserve globally shared structure while adapting to recurring period-specific appearance changes.

To address this problem, we propose a Semantic Prior-Guided Period-Aware Multi-Expert Segmentation framework, termed SPMES, for long-term fixed-view visual monitoring scenes. Instead of treating all images as independent samples from a generic segmentation dataset, SPMES explicitly exploits the monitoring setting: the stable spatial layout is modeled as a global semantic prior, and the recurring appearance variations are modeled by period-specific experts. The framework consists of a Global Expert (GE), a Semantic-Guided Fusion Module (SGFM), and multiple period-specific experts (PSEs). GE is trained on the entire training set to produce stable semantic-prior maps as global structural references. SGFM injects these priors into the input representation through semantic-guided interaction, enabling period-specific features to be enhanced under global structural guidance. PSEs specialize in different recurring acquisition periods according to image capture time, improving period-aware segmentation while preserving structural consistency.

Although SPMES is conceptually related to the Mixture of Experts (MoE) paradigm [9,10], it differs from conventional gated MoE architectures in two aspects. First, expert specialization is explicitly grounded in acquisition-period information provided by the monitoring system, rather than being learned only from ambiguous appearance cues. Second, expert collaboration is constrained by a shared global semantic prior, which helps prevent period-specific appearance modeling from drifting away from the stable scene structure. This design is intended for fixed-view monitoring scenes, where the scene layout remains stable while appearance varies across acquisition periods.

The main contributions of this work are summarized as follows:

- We formulate long-term fixed-view monitoring segmentation as a structure–appearance collaborative modeling problem, where stable scene-level semantic structure and recurring acquisition-period-specific appearance variations need to be jointly considered.
- We propose SPMES, a Semantic Prior-Guided Period-Aware Multi-Expert Segmentation framework. It integrates a Global Expert for learning stable semantic-prior maps, a Semantic-Guided Fusion Module for structure-aware representation fusion, and period-specific experts with deterministic acquisition-period-based routing for appearance-specialized segmentation under global structural guidance.
- We conduct systematic experiments on a long-term fixed-view monitoring dataset collected for this study. Results across multiple representative segmentation backbones, together with sensitivity analysis, ablation studies, qualitative comparisons, and complexity analysis, support the effectiveness of SPMES in the studied deployment setting and its compatibility with different segmentation backbones.

## 2. Related Work

Existing studies have improved semantic segmentation from the perspectives of backbone architectures, feature fusion and attention mechanisms, period-aware scene modeling, and multi-expert systems. However, for long-term fixed-view monitoring scenes, the key challenge is not only to improve generic feature extraction, but also to exploit the coexistence of stable scene structure and acquisition-period-specific appearance variation. This section reviews these research directions and clarifies how SPMES differs from existing approaches.

### 2.1. Backbone Semantic Segmentation Models

Encoder–decoder architectures remain a dominant paradigm in semantic segmentation. Classical CNN-based models such as U-Net [11], U-Net++ [12], and U^2^-Net [13] improve multi-scale semantic modeling and boundary recovery. More recently, Transformer-based segmentation models, including SETR [14], Segmenter [15], SegFormer [16], and Swin-Unet [17], have demonstrated strong representation capabilities. Among them, Swin-Unet is adopted in this study as a representative Transformer-based segmentation backbone. Foundation-model-based approaches [2,4,5] have also shown considerable potential for semantic segmentation. State-space-model-based architectures, such as Mamba-UNet [18,19,20,21,22,23], further provide new alternatives for efficient visual modeling. These models are powerful general-purpose segmentation backbones, but they usually do not explicitly exploit acquisition-period information or fixed-view structural priors. In this work, SPMES is designed as a framework that can be integrated with different backbones to introduce period-aware and semantic-prior-guided modeling.

### 2.2. Feature Fusion and Attention Mechanisms

Feature fusion and attention mechanisms are widely used to improve semantic segmentation by integrating multi-scale semantics and reweighting informative regions [24,25,26]. Recent studies on high-quality segmentation also indicate that preserving boundary and topology consistency is important for reliable segmentation results [27]. However, most existing fusion or attention modules operate within the internal feature space of a single model and do not explicitly use stable scene-level semantic priors from fixed-view monitoring data. In long-term monitoring scenes, local textures, contrast, and boundary visibility may vary substantially across acquisition periods. Under such conditions, purely internal feature interactions may still be affected by appearance changes. Therefore, SPMES introduces a global semantic prior produced by GE and uses SGFM to inject this prior into period-specific representations, enabling appearance modeling to be guided by stable structure.

### 2.3. Period-Aware Scene Modeling

The period-related variations considered in this work are caused by recurring seasonal, illumination, weather, and environmental changes under a fixed viewpoint, rather than by object motion as in video segmentation tasks. Existing studies have addressed temporal and condition-induced appearance variations from different perspectives. Time-aware or stage-aware methods introduce acquisition time as an auxiliary condition for scene modeling [28]. Cross-season semantic segmentation and long-term visual localization methods exploit correspondences between observations captured under different seasons or visual conditions to improve semantic consistency [29,30]. Cross-condition adaptation methods further align or contrast corresponding observations to enhance segmentation robustness under adverse conditions [31,32], while style-adaptive methods address appearance variations from the perspective of domain generalization [8]. These methods generally formulate the problem through temporal conditioning, cross-condition consistency, domain adaptation, or domain generalization, and may rely on explicit source and target domains, unlabeled target-domain data, or corresponding observations captured under different conditions. In contrast, the present work considers fully supervised segmentation within a single fixed-view monitoring scene, where the training images are provided with pixel-level annotations and acquisition timestamps, without constructing explicit source and target domains. The purpose of period-specific learning is therefore to reduce recurring intra-scene appearance heterogeneity while retaining the approximately stable spatial structure shared across different acquisition periods. A straightforward alternative is to train separate models for different periods; however, independently trained models may suffer from limited period-specific samples and insufficient global structural knowledge. This motivates the proposed collaborative design that combines period-specific expert specialization with a shared global semantic prior.

### 2.4. Multi-Expert Systems in Vision Tasks

Mixture of Experts (MoE) improves model flexibility and generalization by assigning complementary expert modules to different data patterns [10,33,34]. This idea is naturally related to long-term fixed-view monitoring scenes, where different acquisition periods correspond to different appearance patterns. However, most existing vision MoE methods rely on learned gates or implicit expert partitioning strategies [34]. Such strategies may introduce additional routing uncertainty and usually do not impose a unified global semantic constraint. By contrast, SPMES adopts deterministic routing based on acquisition periods and introduces a shared global semantic prior to constrain period-specific experts, aiming to preserve both period-specific expressiveness and global structural consistency.

## 3. Methods

In long-term fixed-view monitoring scenes, seasonal, illumination, weather, and environmental changes may cause substantial appearance variations across acquisition periods. These variations affect local textures, contrast, and boundary visibility, leading to degraded segmentation accuracy and unstable boundary prediction.

The design of SPMES is motivated by a practical property of fixed-view visual monitoring systems: although local appearance may change substantially, the camera viewpoint and the global spatial layout of the monitored target usually remain stable. Therefore, segmentation can benefit from a collaborative design that uses global structural knowledge as a stable reference while allowing specialized models to adapt to recurring period-specific appearance patterns.

**Figure 1:**
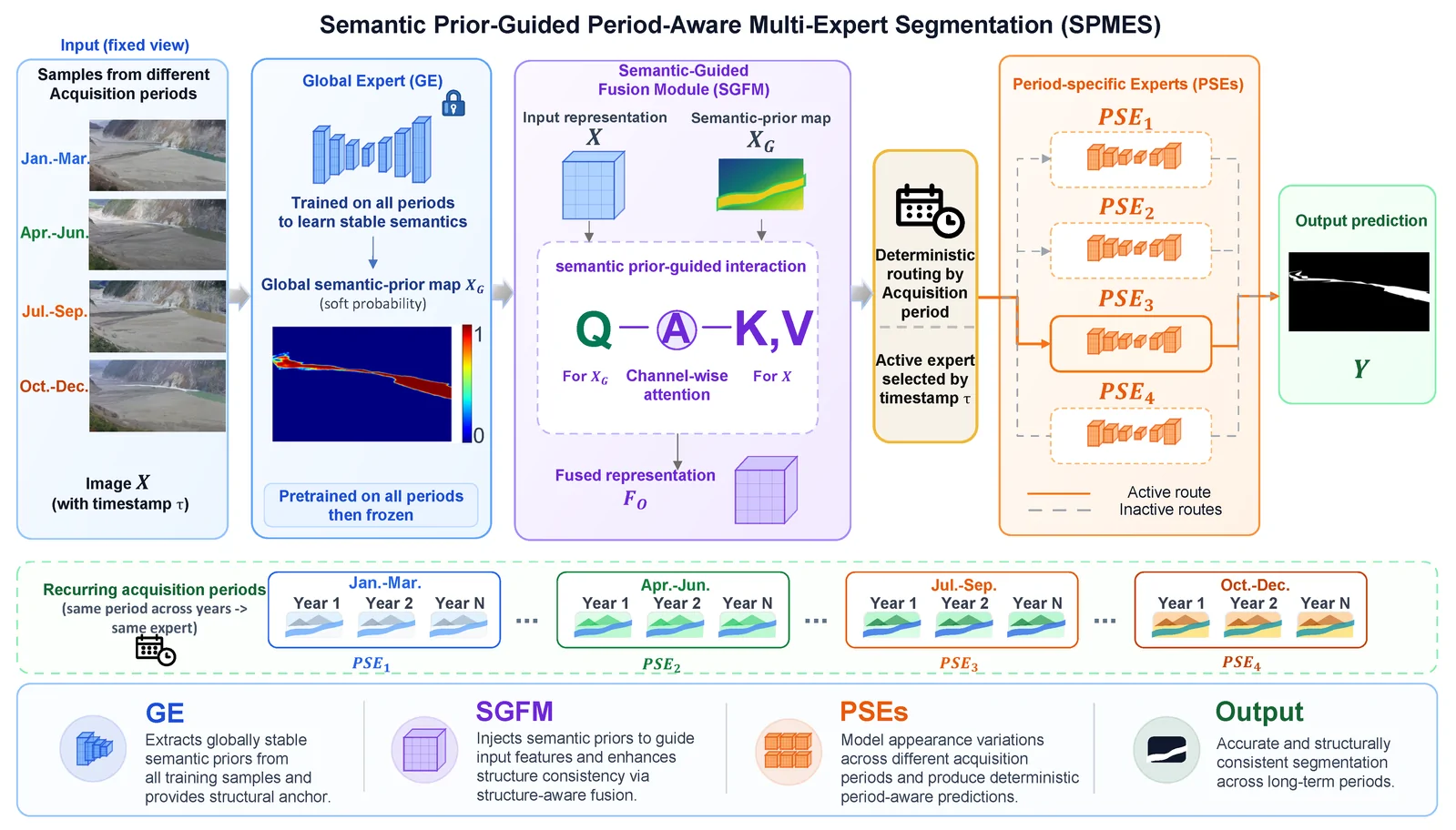
Overview of the proposed SPMES framework for long-term fixed-view monitoring segmentation. The Global Expert (GE) produces stable semantic-prior maps, the Semantic-Guided Fusion Module (SGFM) performs structure-aware representation fusion, and the period-specific experts (PSEs) specialize in recurring acquisition periods for deterministic period-aware prediction.

To this end, we propose a Semantic Prior-Guided Period-Aware Multi-Expert Segmentation framework, termed SPMES. It consists of three components: a Global Expert (GE), a Semantic-Guided Fusion Module (SGFM), and period-specific experts (PSEs). GE learns stable semantic priors from the whole training set and produces soft semantic-prior maps; SGFM injects these priors into the input representation for structure-aware enhancement; and PSEs model period-specific appearance characteristics. The overall architecture is shown in Figure 1. The following subsections describe the overall architecture, the three principal components, the training objective, and the staged training and timestamp-based inference strategy.

### 3.1. Overall Architecture

Given an input image $X\in R^{C\times H^{\prime }\times W^{\prime }}$, SPMES performs semantic segmentation through three stages: global semantic-prior extraction, semantic-guided representation fusion, and period-specific expert prediction. First, GE produces a stable semantic-prior map from the input image. Then, SGFM fuses the global semantic prior with the input representation to obtain a structure-enhanced representation. Finally, the fused representation is routed to the corresponding period-specific expert according to the acquisition period.

The overall process is formulated as:

(1) $$\begin{array}{cc} X_{G} & =GE(X), \end{array}$$

(2) $$\begin{array}{cc} F_{O} & =SGFM(X,X_{G}), \end{array}$$

(3) $$\begin{array}{cc} Y & =M_{t}\left(F_{O}\right), \end{array}$$

where $X_{G}$ denotes the global semantic-prior map, $F_{O}$ denotes the fused structure-enhanced representation, $M_{t}$ denotes the expert selected according to the acquisition period of the input image, and *Y* denotes the final prediction.

The framework explicitly coordinates globally shared structural information and period-specific appearance information. GE models stable semantic structure from all training samples, PSEs model acquisition-period-specific appearance patterns, and SGFM establishes representation-level interaction between them. SPMES can be integrated with different backbones, including U-Net, U-Net++, U^2^-Net^†^, Swin-Unet, and Mamba-UNet.

### 3.2. Global Expert

The Global Expert (GE) is designed to learn globally shared semantic structure and provide a stable semantic-prior reference for subsequent period-specific representation learning. In long-term fixed-view monitoring scenarios, although local appearance may change significantly due to seasonal, illumination, weather, and environmental variations, the overall spatial layout of the monitored target is usually relatively stable. For example, in water-body monitoring, local water boundaries and surface textures may fluctuate across periods, whereas the overall spatial distribution of the water region is usually more consistent. Therefore, it is desirable to introduce an independent module to model such stable structure shared by samples from different acquisition periods.

In implementation, GE adopts the same network architecture as the selected backbone and is pretrained on the entire training set to capture semantic structural patterns shared by all periods. Unlike the period-specific experts, GE is not optimized for a specific acquisition period. Instead, it extracts globally shared structural information from the overall data distribution and thereby forms a stable global semantic prior.

During subsequent training stages, the parameters of GE are kept frozen. The main purpose of this design is to use GE as a stable semantic-prior extractor, preventing it from being biased toward any specific acquisition period during the optimization of period-specific experts. If GE were jointly updated with different period-specific experts, its representation might gradually drift toward local period-specific appearance patterns, thereby weakening its role as a global structural reference. By contrast, freezing GE ensures that all period-specific experts refer to the same global semantic basis, which helps improve structural consistency and reduce overfitting caused by limited period-specific samples.

As defined in Equation (1), GE outputs the global semantic-prior map $X_{G}\in R^{C_{G}\times H^{\prime }\times W^{\prime }}$. For binary water-body segmentation, $C_{G}=1$.

In practice, $X_{G}$ denotes the soft semantic probability map produced by GE before thresholding, rather than the binarized segmentation mask. Therefore, $X_{G}$ preserves continuous confidence information and provides a soft structural prior for subsequent semantic-guided fusion.

Since this prior map is learned from samples across all acquisition periods, it provides a stable structural reference for subsequent modules and helps reduce the influence of period-specific illumination, seasonal, and environmental variations on feature learning.

### 3.3. Period-Specific Experts

Unlike the relatively stable semantic structure shared across acquisition periods, local appearance in long-term monitoring data often exhibits clear period-specific differences. For instance, seasonal changes may alter vegetation coverage, water boundaries, or background textures, while illumination variation directly affects brightness, contrast, and local texture patterns. To better model such period-related appearance variation, we introduce multiple period-specific experts (PSEs), each dedicated to a specific acquisition period.

Before period-specific optimization, the parameters of the pretrained Global Expert are copied to initialize all period-specific experts. After initialization, the experts do not share parameters with the Global Expert or with one another, and each expert is further optimized using only the training samples belonging to its corresponding acquisition period.

Specifically, based on the acquisition time of each image within the annual cycle, the annual acquisition cycle is uniformly divided into *T* recurring acquisition periods, and an expert model $\left\{M_{1},M_{2},\ldots ,M_{T}\right\}$ is assigned to each period. For example, when T=4, the annual cycle is divided into four periods: from January to March, from April to June, from July to September, and from October to December. Samples acquired during the same period in different years are assigned to the same expert.

The uniform partition is intended as a simple and reproducible routing approximation rather than an exact representation of natural-season or hydrological boundaries. Such transitions are gradual and may vary across regions and years, while precipitation, runoff, snowmelt, and episodic ice-avalanche events can cause irregular changes that cannot be inferred reliably from timestamps alone. We therefore adopt calendar-based routing without requiring additional environmental metadata and evaluate its temporal granularity through the sensitivity analysis of *T*.

This partitioning strategy is designed to capture recurring seasonal, illumination-related, and environmental appearance patterns while keeping the routing process deterministic. Each period-specific expert adopts the same architecture as the selected backbone, but different experts do not share parameters and are trained only on samples belonging to their corresponding periods. Consequently, each expert focuses on a relatively narrower range of appearance variations rather than fitting all heterogeneous long-term conditions within a single parameter space.

For a fused representation $F_{O}$ with acquisition time τ, the sample is routed to expert $M_{t}$ when $\tau \in S_{t}$, where $S_{t}$ denotes the *t*-th recurring acquisition period. The final prediction is then obtained according to Equation (3).

Because timestamps directly indicate the acquisition period to which a sample belongs, we adopt a deterministic routing strategy based on image capture time instead of introducing an additional gating network. This design deterministically assigns each sample to the corresponding period-specific expert while avoiding the additional uncertainty and training complexity associated with learned gating.

From the perspective of appearance modeling, samples from different acquisition periods contain distinct visual characteristics. If a single model is used to fit all periods jointly, it must represent multiple heterogeneous appearance patterns within the same parameter space, which may lead to performance trade-offs across periods. In contrast, the period-aware multi-expert design decomposes the overall learning problem into several relatively simpler period-specific subproblems, thereby improving appearance representation while GE and SGFM provide global structural guidance.

### 3.4. Semantic-Guided Fusion Module

**Figure 2:**
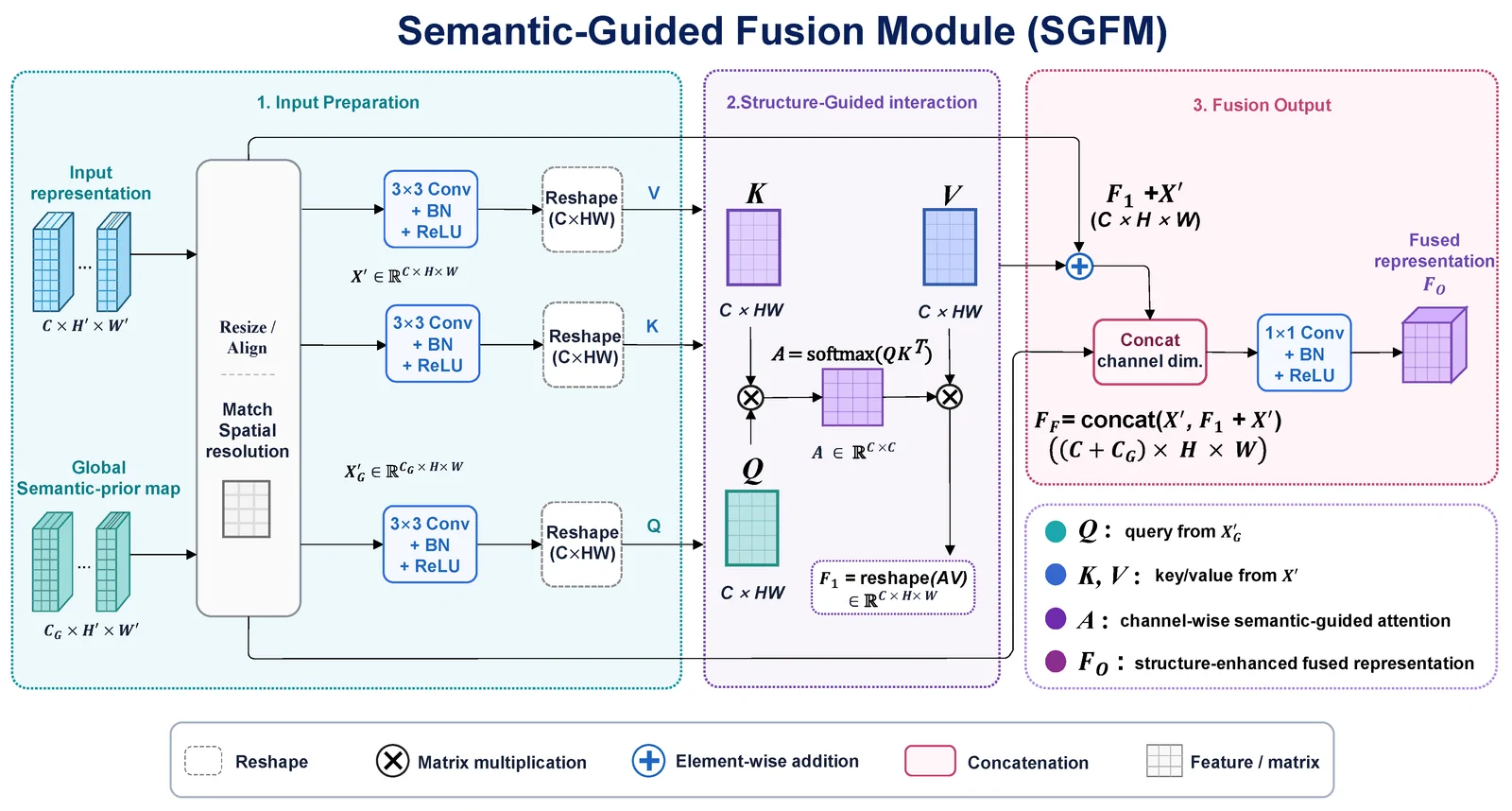
Architecture of the Semantic-Guided Fusion Module (SGFM). The Value and Key are generated from the input representation, whereas the Query is generated from the global semantic-prior map.

To effectively inject globally stable semantic structure into the period-specific input representation, we design a Semantic-Guided Fusion Module (SGFM), as illustrated in Figure 2. This module establishes interactions between the input representation and the global semantic-prior map through a prior-guided channel-attention mechanism for cross-feature fusion, so that stable structural information can guide period-specific appearance features while enhancing the representation of key target regions.

The inputs of SGFM include the input image tensor $X\in R^{C\times H^{\prime }\times W^{\prime }}$ and the global semantic-prior map $X_{G}\in R^{C_{G}\times H^{\prime }\times W^{\prime }}$ produced by GE. Since the two inputs need to share the same spatial scale before fusion, they are first downsampled to a unified spatial resolution:

$$X^{\prime }\in R^{C\times H\times W},\;X_{G}^{\prime }\in R^{C_{G}\times H\times W}.$$

Although $X_{G}^{\prime }$ and $X^{\prime }$ have $C_{G}$ and *C* channels, respectively, $C_{G}$ is not required to be equal to *C*. The Query projection ${\mathrm{Conv}}_{Q}$ maps the global semantic-prior representation from $C_{G}$ channels to *C* channels, whereas ${\mathrm{Conv}}_{K}$ and ${\mathrm{Conv}}_{V}$ preserve the *C*-channel dimension of the input representation. For the binary segmentation task considered in this work, $C_{G}=1$, and ${\mathrm{Conv}}_{Q}$ projects the single-channel soft semantic prior into *C* Query channels. Then, convolution, normalization, and nonlinear activation are applied to generate the Value, Key, and Query representations, which are reshaped into matrix form:

(4) $$\begin{array}{cc} V & =\mathrm{reshape}\;\left(\mathrm{ReLU}\;\left({\mathrm{BN}}_{V}\;\left({\mathrm{Conv}}_{V}\left(X^{\prime }\right)\right)\right)\right)\in R^{C\times (H\times W)}, \end{array}$$

(5) $$\begin{array}{cc} K & =\mathrm{reshape}\;\left(\mathrm{ReLU}\;\left({\mathrm{BN}}_{K}\;\left({\mathrm{Conv}}_{K}\left(X^{\prime }\right)\right)\right)\right)\in R^{C\times (H\times W)}, \end{array}$$

(6) $$\begin{array}{cc} Q & =\mathrm{reshape}\;\left(\mathrm{ReLU}\;\left({\mathrm{BN}}_{Q}\;\left({\mathrm{Conv}}_{Q}\left(X_{G}^{\prime }\right)\right)\right)\right)\in R^{C\times (H\times W)}. \end{array}$$

Here, ${\mathrm{Conv}}_{V}$, ${\mathrm{Conv}}_{K}$, and ${\mathrm{Conv}}_{Q}$ are independent 3×3 convolution layers that generate channel-aligned Value, Key, and Query representations, and ${\mathrm{BN}}_{V}$, ${\mathrm{BN}}_{K}$, and ${\mathrm{BN}}_{Q}$ are the corresponding independent batch-normalization layers. These projections constitute the initial projection stage within the SGFM operation $F_{O}=SGFM\left(X,X_{G}\right)$ defined in Equation (2).

Different from standard self-attention, SGFM uses the global semantic-prior map $X_{G}^{\prime }$ to generate Query, while Key and Value are generated from the input representation $X^{\prime }$. The core idea of this design is to use stable semantic structural priors to reweight channel responses in the input representation, thereby enhancing target-related responses. In other words, through a structure–query–appearance–response mechanism, the model is encouraged to focus on regions that are more consistent with the global semantic structure under dynamic appearance conditions.

Based on these representations, the channel-wise attention matrix is computed as:

(7) $$\begin{array}{c} A=\mathrm{softmax}\left(QK^{⊤}\right)\in R^{C\times C}, \end{array}$$

where softmax(·) normalizes the channel correlations to obtain feature-reweighting coefficients guided by the global semantic prior. The channel-wise formulation is adopted because the input representation and the GE-generated semantic prior are spatially aligned in the fixed-view setting. SGFM therefore focuses on modulating semantic-related channel responses, while the residual pathway introduced below preserves the original spatial information. Moreover, the proposed formulation constructs a C×C attention matrix, whereas full pairwise spatial attention would construct an (HW)×(HW) matrix, with computational complexities of $O(C^{2}HW)$ and $O(C{\left(HW\right)}^{2})$, respectively. This design reduces the memory and computational cost of the fusion operation relative to full pairwise spatial attention.

Next, the attention matrix is used to weight the Value feature, and the resulting feature is reshaped back into the spatial form:

(8) $$\begin{array}{c} F_{1}=\mathrm{reshape}\left(AV\right)\in R^{C\times H\times W}. \end{array}$$

To preserve local appearance details while introducing structure-enhanced representations, we first construct the fused representation by

(9) $$\begin{array}{c} F_{F}=\mathrm{concat}\;\left(X_{G}^{\prime },\;F_{1}+X^{\prime }\right)\in R^{(C+C_{G})\times H\times W}. \end{array}$$

Since the concatenation increases the channel dimension, a projection layer is further introduced to perform channel alignment and feature adaptation:

(10) $$\begin{array}{c} F_{O}=\mathrm{ReLU}\;\left({\mathrm{BN}}_{F}\;\left({\mathrm{Conv}}_{1\times 1}\left(F_{F}\right)\right)\right)\in R^{C\times H\times W}. \end{array}$$

Here, the residual term $F_{1}+X^{\prime }$ helps retain local appearance details from the input representation while injecting semantic-guided information, whereas concatenation with $X_{G}^{\prime }$ explicitly preserves the global semantic structure. The projection layer, implemented by ${\mathrm{Conv}}_{1\times 1}$ followed by ${\mathrm{BN}}_{F}$ and ReLU, further performs feature mixing and dimensional alignment, enabling the fused representation to be consistently adapted to the input interface of the period-specific expert. In this way, SGFM enables collaborative fusion of global structural priors and local appearance representations.

Overall, unlike conventional attention mechanisms that rely solely on internal feature interactions, SGFM introduces global semantic priors to establish guided interactions across representation spaces. This design enhances responses in key structural regions, suppresses background noise and semantic ambiguity under complex visual conditions, and improves structural consistency for period-specific expert prediction.

For clarity, the principal symbols and tensor dimensions used in the preceding formulation are summarized in Table 1.

### 3.5. Loss Function

To improve both pixel-wise classification accuracy and region-level overlap consistency, we adopt a weighted combination of Binary Cross-Entropy (BCE) loss and Dice loss as the training objective.

The BCE loss is defined as:

(11) $$\begin{array}{c} L_{BCE}=-\frac{1}{N}\sum_{i=1}^{N}\left[g_{i}\log\left(p_{i}\right)+\left(1-g_{i}\right)\log\left(1-p_{i}\right)\right], \end{array}$$

where $p_{i}$ denotes the predicted probability that pixel *i* belongs to the foreground class, $g_{i}$ denotes the corresponding ground-truth label, and *N* denotes the total number of pixels in the image.

Dice loss is used to measure the overlap between the predicted region and the ground-truth annotation, and is defined as:

(12) $$\begin{array}{c} L_{Dice}=1-\frac{2\sum_{i=1}^{N}p_{i}g_{i}+\epsilon }{\sum_{i=1}^{N}p_{i}+\sum_{i=1}^{N}g_{i}+\epsilon }, \end{array}$$

where ϵ is a smoothing term to avoid division by zero.

The final training loss is formulated as:

(13) $$\begin{array}{c} L=\alpha L_{BCE}+\beta L_{Dice}, \end{array}$$

where α and β denote the weighting coefficients of BCE loss and Dice loss, respectively. The ratio α:β controls the relative contributions of pixel-wise classification supervision and region-overlap optimization. A larger α places greater emphasis on pixel-wise classification, whereas a larger β increases the contribution of region-level overlap consistency. In the main experiments, α:β was set to 5:1, as selected through the validation-set sensitivity analysis presented in Section 4.4.1.

### 3.6. Training and Inference Strategy

SPMES is optimized in two stages. GE is first trained on the complete training set and then frozen, while its parameters are copied to initialize all PSEs. During the second stage, each sample is deterministically routed to its corresponding PSE according to the acquisition timestamp; the shared SGFM and the routed PSE are optimized, whereas GE remains fixed. During inference, the timestamp selects one PSE, and GE, SGFM, and the selected PSE sequentially produce the final prediction. The complete procedure is summarized in Algorithm 1.
**Algorithm 1** Staged training and inference of SPMES**Require:** Training set $D={\left\{\left(X_{i},Y_{i},\tau_{i}\right)\right\}}_{i=1}^{N}$; number of periods *T*; period-assignment function π(τ;T); segmentation loss L**Ensure:** Trained GE parameters $\theta_{G}$, shared SGFM parameters ϕ, and PSE parameters $\left\{\theta_{1},\ldots ,\theta_{T}\right\}$  1:**Stage 1: Global Expert pretraining**  2:Initialize GE parameters $\theta_{G}$  3:**for** each GE training epoch **do**  4:    **for** each mini-batch B⊂D **do**  5:        Let *X* and *Y* denote the input images and labels in B  6:        ${\hat{Y}}_{G}\leftarrow GE\left(X;\theta_{G}\right)$  7:        $L_{G}\leftarrow L\left({\hat{Y}}_{G},Y\right)$  8:        Update $\theta_{G}$ by minimizing $L_{G}$  9:    **end for** 10:**end for** 11:**Stage 2: Period-specific initialization and optimization** 12:**for** t=1,…,T **do** 13:    Initialize the *t*-th PSE by $\theta_{t}\leftarrow \theta_{G}$ 14:**end for** 15:Freeze $\theta_{G}$ 16:Initialize the shared SGFM parameters ϕ 17:**for** each period-specific training epoch **do** 18:    **for** each mini-batch B⊂D **do** 19:        $L_{B}\leftarrow 0$ 20:        **for** t=1,…,T **do** 21:           $B_{t}\leftarrow \left\{\left(X_{i},Y_{i},\tau_{i}\right)\in B|\pi \left(\tau_{i};T\right)=t\right\}$ 22:           **if** $B_{t}\neq ⌀$ **then** 23:               Let $X_{t}$ and $Y_{t}$ denote the input images and labels in $B_{t}$ 24:               $X_{G}\leftarrow \mathrm{StopGrad}\left(GE(X_{t};\theta_{G})\right)$ 25:               $F_{O}\leftarrow SGFM\left(X_{t},X_{G};\phi \right)$ 26:               ${\hat{Y}}_{t}\leftarrow M_{t}\left(F_{O};\theta_{t}\right)$ 27:               $L_{B}\leftarrow L_{B}+L\left({\hat{Y}}_{t},Y_{t}\right)$ 28:           **end if** 29:        **end for** 30:        Update ϕ and the active parameters among ${\left\{\theta_{t}\right\}}_{t=1}^{T}$ by minimizing $L_{B}$; keep $\theta_{G}$ fixed 31:    **end for** 32:**end for** 33:**Inference for an image *X* acquired at time τ** 34:$t^{*}\leftarrow \pi \left(\tau ;T\right)$ 35:Select the corresponding expert $M_{t^{*}}$ 36:$X_{G}\leftarrow GE\left(X;\theta_{G}\right)$ 37:$F_{O}\leftarrow SGFM\left(X,X_{G};\phi \right)$ 38:$\hat{Y}\leftarrow M_{t^{*}}\left(F_{O};\theta_{t^{*}}\right)$ 39:**return** $\hat{Y}$

## 4. Experiments and Results

This section first describes the dataset, experimental settings, and evaluation metrics. We then report validation-based sensitivity analyses of the loss-weight ratio and the number of acquisition periods, followed by comparisons across five segmentation backbones and direct comparisons with representative period-aware baselines. A focused repeated-seed analysis with paired confidence intervals is further conducted for representative Swin-Unet configurations. Additional experiments examine the effect of input resolution, followed by qualitative comparisons and component ablations. Finally, model complexity, inference-time resource requirements, and the deployment implications and limitations of SPMES in the studied low-frequency server-side monitoring setting are analyzed.

### 4.1. Dataset

The dataset was collected by a long-term visual monitoring system deployed at an ice-avalanche-induced river blockage site. A fixed-position camera repeatedly observes the same scene, with images acquired approximately once per hour and transmitted through a satellite link to a central server, where the segmentation model is currently executed.

The raw image collection spans from April 2021 to October 2023 and contains 7231 RGB images with a resolution of 2560×1440 in JPG format. After corrupted or low-quality images were removed, representative samples were selected across the full annotated interval and manually annotated to provide long-term temporal coverage. The annotated images used in the experiments were acquired from 19 April 2021 to 30 April 2023, covering approximately two annual acquisition cycles.

The segmentation task was formulated as binary pixel-level classification, with the visually identifiable water surface treated as the foreground class and all non-water regions treated as background. The water-region boundary was delineated along the visible interface between the water surface and adjacent non-water regions.

The 1360 masks were manually annotated at the original image resolution using LabelMe [35] by two annotators following the same class and boundary criteria. Each completed annotation was cross-checked by the other annotator. Ambiguous boundaries or inconsistent labels were jointly reviewed and corrected by consensus before the final annotation was determined. The resulting LabelMe annotations were subsequently converted into binary segmentation masks for model training and evaluation. Because the final masks were obtained through consensus correction rather than retained as two separate independent annotations, quantitative inter-annotator agreement statistics are not available.

Representative images and their ground-truth masks are provided in the qualitative comparisons presented later. Together with the annotation criteria and quality-control procedure described above, these examples provide a reference for understanding the annotation process.

The final annotated dataset contains 1025 training images, 100 validation images, and 235 test images. The dataset split was performed at the calendar-day level before acquisition-period labels were assigned. All images acquired on the same calendar day were treated as one date group and assigned exclusively to the training, validation, or test set. Therefore, observations from the same daily acquisition sequence were not distributed across different subsets. For each subset, the selected date groups were distributed approximately uniformly over the full annotated interval rather than being concentrated within a short temporal segment. Consequently, the training, validation, and test sets all contain observations acquired throughout the long-term monitoring interval. No explicit categorization or balancing according to illumination, weather, environmental conditions, or the subsequently defined acquisition periods was performed during dataset splitting.

The purpose of period partitioning in this work is not future forecasting or chronological generalization. Instead, acquisition time is used as auxiliary metadata to divide the long-term monitoring samples into several recurring appearance-related periods within the annual acquisition cycle, thereby enabling period-specific expert specialization. All annotated images were acquired using the same fixed-position monitoring device at a single monitoring site. The camera viewpoint remained stable throughout the data-collection period; therefore, no additional image registration or stabilization was applied during preprocessing. The dataset supports evaluation of recurring long-term appearance variations represented within this deployment, but it does not directly evaluate chronological, cross-camera, or cross-site generalization.

Most publicly available semantic segmentation datasets, such as Cityscapes [36] and ADE20K [37], emphasize spatial semantic diversity rather than long-term repeated observation of the same scene from a fixed viewpoint. By contrast, our dataset provides a fixed observation viewpoint, a monitoring duration of more than two years, and substantial variation in illumination, weather conditions, and water-body morphology. These characteristics make it suitable for studying period-aware segmentation in long-term fixed-view monitoring scenes, where stable spatial layout and period-specific appearance variation coexist. Since the proposed expert specialization relies on recurring acquisition periods under a stable viewpoint, conventional public segmentation datasets without long-term repeated observations and acquisition-time metadata are not directly suitable for evaluating this setting.

### 4.2. Experimental Settings

All experiments were conducted under a unified hardware and software environment. The hardware configuration includes an Intel Xeon Gold 6430 CPU, 32 GB memory, and one NVIDIA A30 GPU with 24 GB memory. The software environment consists of Debian 12, Python 3.11, PyTorch 2.5.1, and CUDA 11.8.

During training, the Adam optimizer was adopted with an initial learning rate of $1\times 10^{-4}$, and cosine annealing was used for learning-rate scheduling. The maximum number of training epochs was set to 300, and the batch size was 2. Unless otherwise stated, all input images were resized to 320×320. To examine the possible loss of thin boundary information caused by spatial downsampling, an additional controlled input-resolution analysis was conducted using 320×320, 480×480, and 640×640, as described in Section 4.7. Strong geometric augmentations, such as random cropping and random rotation, were not applied because the stable fixed-view setting provides useful spatial correspondence between image coordinates and scene structures.

Given the relatively limited number of annotated training images, several measures were adopted to mitigate overfitting and prevent optimistic evaluation. First, the dataset was split at the calendar-day level, such that all images acquired on the same day were assigned exclusively to one subset. This reduces the possibility that highly similar observations from the same daily acquisition sequence appear in both the training and evaluation sets. Second, model and hyperparameter selection were performed using the validation set, while the test set was reserved for final evaluation.

For SPMES, the period-specific experts were not trained from random initialization using only their relatively small period-specific subsets. Instead, GE was first trained using all 1025 training images, and its parameters were then copied to initialize each PSE. During subsequent period-specific optimization, GE was frozen and provided a common semantic prior, while the shared SGFM received training samples from all acquisition periods. This staged initialization and shared structural guidance reduce the number of period-specific patterns that must be learned from scratch. Cosine learning-rate annealing was also used to stabilize optimization. These measures mitigate, but do not completely eliminate, the risk of overfitting associated with the limited and scene-specific dataset.

To examine the compatibility of the proposed framework with different segmentation architectures, five representative semantic segmentation backbones were selected as baselines, namely U-Net [11], U-Net++ [12], U^2^-Net^†^ [13], Swin-Unet [17], and Mamba-UNet [18]. For each backbone, we trained both the original baseline model and its SPMES-enhanced counterpart under identical experimental settings. Here, U^2^-Net^†^ corresponds to the u2netp implementation released in the official U^2^-Net repository.

For the loss setting, the weighted combination of BCE loss and Dice loss defined in Section 3.5 was used as the training objective. The ratio α:β=5:1 was selected using the Swin-Unet-based GE validation analysis in Section 4.4.1 and was kept unchanged in the subsequent experiments.

For fair comparison, all baseline models and their SPMES-enhanced counterparts were trained and evaluated using the same train/validation/test split and preprocessing pipeline described in Section 4.1.

Unless otherwise specified, the number of acquisition periods was set to T=4, which was selected exclusively through the validation-set sensitivity analysis described in Section 4.4.2. Candidate values T∈{2,3,4,5} were compared using the same training and validation sets, without using the test set for model selection. After T=4 was fixed, the test set was used only for final performance evaluation. Under this setting, the annual acquisition cycle was divided into four periods: January to March, April to June, July to September, and October to December. For SPMES, the acquisition-period labels were derived directly from image timestamps and used only for deterministic expert routing. The corresponding period-wise distributions of the training, validation, and test sets are reported in Table 2.

The five-backbone comparison, most component ablations, the loss-weight analysis, and the input-resolution analysis were conducted once per configuration and are therefore interpreted descriptively. Complete end-to-end repeated training was limited to four representative Swin-Unet-based configurations using seeds 3407, 2026, and 42, as detailed in Section 4.6.

### 4.3. Evaluation Metrics

To comprehensively evaluate segmentation performance from the perspectives of region overlap, boundary accuracy, and shape preservation, we adopt IoU, Dice, Recall, Boundary F1-score (BF1), 95% Hausdorff Distance (HD_95_), and Perimeter Error (PE) as evaluation metrics.

IoU, Recall, and Dice are defined as follows:

(14) $$\begin{array}{cc} \mathrm{IoU} & =\frac{TP}{TP+FP+FN}, \end{array}$$

(15) $$\begin{array}{cc} \mathrm{Recall} & =\frac{TP}{TP+FN}, \end{array}$$

(16) $$\begin{array}{cc} \mathrm{Dice} & =\frac{2TP}{2TP+FP+FN}. \end{array}$$

where TP, FP, and FN denote the numbers of true-positive, false-positive, and false-negative pixels, respectively.

In addition to region-based metrics, we also introduce BF1, HD_95_, and PE to evaluate the consistency between predicted and ground-truth boundaries as well as overall shape error. BF1 is computed as:

(17) $$\begin{array}{cc} P_{b} & =\frac{\left|\left\{\;p\in B_{P}|d\left(p,B_{G}\right)\leq r\;\right\}\right|}{|B_{P}|}, \end{array}$$

(18) $$\begin{array}{cc} R_{b} & =\frac{\left|\left\{\;g\in B_{G}|d\left(g,B_{P}\right)\leq r\;\right\}\right|}{|B_{G}|}, \end{array}$$

(19) $$\begin{array}{cc} \mathrm{BF}1 & =\frac{2P_{b}R_{b}}{P_{b}+R_{b}}. \end{array}$$

HD_95_ is defined as:

(20) $$\begin{array}{c} {\mathrm{HD}}_{95}=\max\left\{{\mathrm{Percentile}}_{95}\;\left(d(B_{P},B_{G})\right),\;{\mathrm{Percentile}}_{95}\;\left(d(B_{G},B_{P})\right)\right\}, \end{array}$$

and PE is defined as:

(21) $$\begin{array}{c} \mathrm{PE}=\frac{\left|\;\mathrm{Perimeter}(P)-\mathrm{Perimeter}(G)\;\right|}{\mathrm{Perimeter}(G)}. \end{array}$$

Here, r=11 denotes the pixel-level distance tolerance, and $B_{P}$ and $B_{G}$ denote the predicted and ground-truth boundary sets, respectively. Before computing boundary-based metrics, the predicted masks are resized back to the original image resolution. Therefore, both the BF1 distance tolerance *r* and the HD_95_ distance values are measured in pixels at the original resolution. For tabular presentation, IoU, Dice, Recall, BF1, and PE are multiplied by 100 and reported as percentages, whereas HD_95_ is reported directly in pixels. Overall, IoU, Dice, and Recall mainly reflect region-level segmentation quality, while BF1, HD_95_, and PE focus more on boundary continuity and shape consistency. Therefore, the combination of these metrics provides a more comprehensive evaluation of segmentation accuracy, boundary continuity, and shape preservation in long-term fixed-view monitoring scenes.

### 4.4. Hyperparameter Sensitivity Analysis

#### 4.4.1. Loss-Weight Ratio

The loss-weight ratio was determined before selecting the number of acquisition periods. Because the BCE-to-Dice weighting is independent of the period partition and is shared by the GE and PSE training stages, we first evaluated α:β∈{1:5,1:3,1:1,3:1,5:1,7:1}, using the Swin-Unet-based Global Expert as the representative segmentation model. The GE was trained using the complete training set without period-specific partitioning, and the candidate ratios were compared exclusively on the validation set. All other training settings, including the data split, optimizer, learning-rate schedule, and model-selection protocol, were kept unchanged. The test set was not used for selecting the loss weights.

As shown in Table 3, the validation performance varies non-monotonically with the BCE-to-Dice loss-weight ratio. The Dice-dominant setting of 1:5 obtains relatively high Recall and BF1 values, but produces the lowest IoU of 79.16%. Increasing the relative contribution of BCE generally improves IoU within the tested range, although small fluctuations are observed between adjacent settings. The highest IoU of 81.52% is obtained at α:β=7:1.

The 5:1 setting achieves the highest Recall of 83.26% and the highest BF1 of 71.63%, while maintaining an IoU of 81.24%. Compared with 7:1, it sacrifices only 0.28 percentage points in IoU, while improving Recall and BF1 by 0.68 and 1.45 percentage points, respectively. It also outperforms the Dice-dominant 1:5 setting across all three reported metrics. Considering the overall balance among region-overlap accuracy, foreground coverage, and boundary quality, α:β=5:1 was retained for the subsequent experiments. Because each candidate ratio was evaluated using a single training run, these results are interpreted as descriptive validation-set comparisons rather than statistical estimates.

#### 4.4.2. Number of Acquisition Periods

To determine the number of acquisition periods without involving the test set, Swin-Unet was selected as the representative backbone and evaluated with T∈{2,3,4,5}. This range covers relatively coarse temporal partitions, the quarterly partition adopted in the final configuration, and a finer five-period partition.

**Figure 3:**
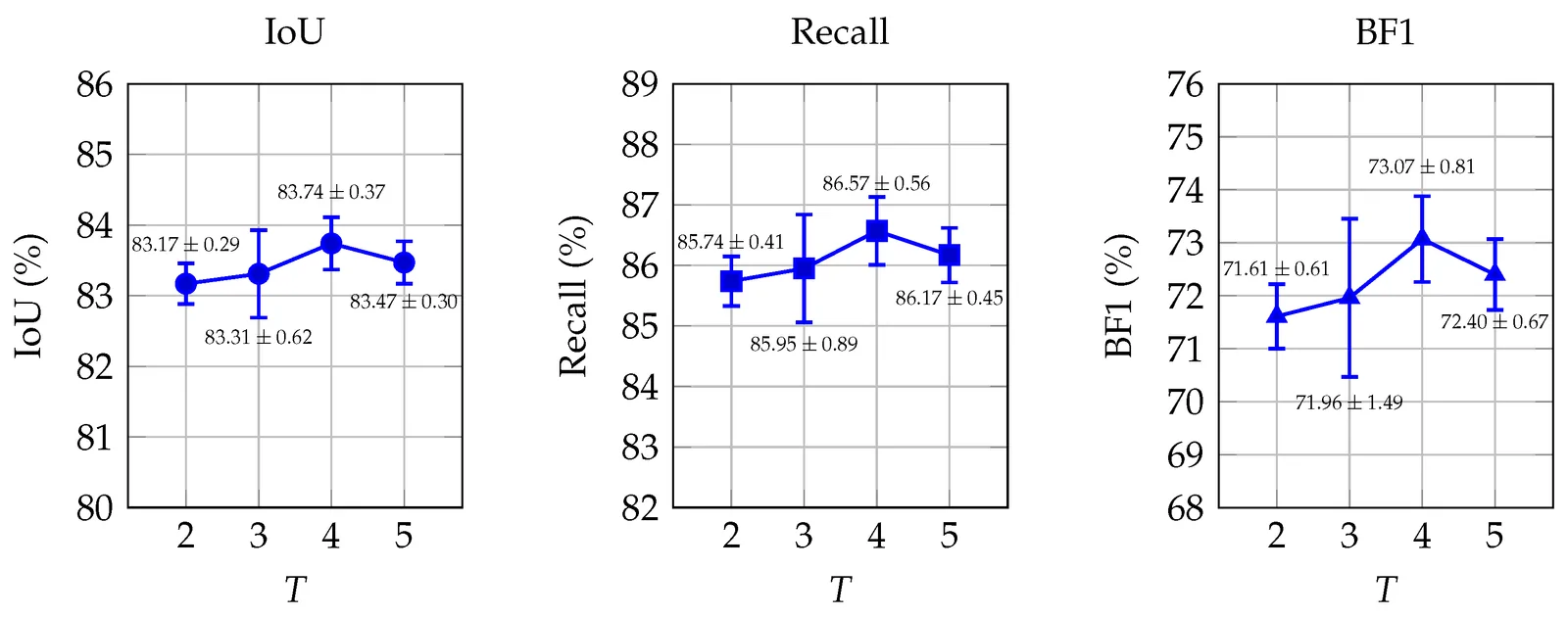
Validation sensitivity to the number of acquisition periods using Swin-Unet. Each point reports mean ± sample standard deviation over three repetitions of the period-specific stage using the same frozen GE checkpoint.

Using the same pretrained and frozen GE checkpoint, the period-specific stage was repeated three times for each T∈{2,3,4,5}. Figure 3 reports the validation IoU, Recall, and BF1 as mean ± sample standard deviation. Accordingly, the reported variability reflects run-to-run variation in the period-specific optimization stage conditional on the fixed GE checkpoint, rather than variability across complete end-to-end retraining of SPMES.

The highest mean values are obtained at T=4, although the differences among neighboring settings are modest. We therefore selected T=4 from the validation results before test evaluation and interpret it as a dataset-specific choice within the examined range rather than a universal temporal partition.

### 4.5. Comparison with Baseline Methods

To evaluate the compatibility of SPMES with different network architectures, five representative segmentation models were selected, including U-Net, U-Net++, U^2^-Net^†^, Swin-Unet, and Mamba-UNet. Here, U^2^-Net^†^ corresponds to the u2netp implementation released in the official U^2^-Net repository. These models cover classical CNN-based architectures, Transformer-based segmentation networks, and state-space-model-based segmentation networks. This design examines whether the observed improvements are tied to a single backbone or remain consistent across the evaluated architectures under the studied monitoring setting. The experimental results are reported in Table 4.

As shown in Table 4, SPMES yields better point estimates across all six reported metrics for the five evaluated backbones on the presented dataset. However, the magnitude of these differences varies substantially across backbones and metrics. For U-Net, relatively clear gains are observed in IoU, Recall, BF1, HD_95_, and PE. For Swin-Unet, PE decreases from 9.08% to 8.28%, corresponding to a modest reduction of 0.80 percentage points, whereas the changes in BF1 and HD_95_ are more pronounced, with BF1 increasing from 68.39% to 72.66% and HD_95_ decreasing from 81.50 to 72.63 pixels.

Taken together, the five-backbone results show a consistent favorable direction in the reported point estimates, although the magnitude of the differences varies across architectures and metrics. Because the results in Table 4 were obtained from one run per configuration, they are interpreted descriptively and should not be regarded as evidence of statistical significance or uniformly large improvements across all backbones and metrics.

### 4.6. Comparison with Temporal Conditioning and Period-Specific Expert Baselines

To determine whether the improvement arises merely from using acquisition-period information, SPMES is compared with Timestamp Conditioning and Period-wise Fine-Tuning (PFT) using Swin-Unet. For this dedicated repeated-run analysis, Swin-Unet, Timestamp Conditioning, PFT, and Swin-Unet with SPMES were independently trained using seeds 3407, 2026, and 42 under the same train/validation/test split, preprocessing pipeline, augmentation strategy, optimization settings, and validation-based model-selection protocol. The results in Table 5 are reported as the mean and sample standard deviation over the three runs.

Timestamp Conditioning maps the acquisition-period index to a learnable embedding and adds it to the bottleneck representation of one shared Swin-Unet. PFT initializes period-specific experts from a GE trained on the complete training set and independently fine-tunes each expert using only its corresponding period. During inference, the timestamp deterministically selects one PFT expert; the original GE prior branch and SGFM are not retained. PFT is therefore a controlled hard-routed period-specific expert baseline rather than a conventional learned-gating mixture-of-experts model.

Because the same three seed identifiers were used for all four configurations, the statistical uncertainty reported in Table 6 was evaluated using paired seed-level differences. For IoU, Dice, Recall, and BF1, the paired difference was defined as the SPMES result minus the comparison result. For HD_95_ and PE, it was defined as the comparison result minus the SPMES result, so that a positive value consistently indicates better performance for SPMES. For each metric, the two-sided 95% confidence interval of the mean paired difference was calculated as

(22) $$\begin{array}{c} \bar{d}\pm t_{0.975,n-1}\frac{s_{d}}{\sqrt{n}}, \end{array}$$

where n=3, $\bar{d}$ is the mean paired difference, and $s_{d}$ is its sample standard deviation. An improvement was regarded as statistically supported for an individual metric when the corresponding interval excluded zero.

This paired analysis was restricted to the four Swin-Unet-based configurations evaluated in this subsection. The five-backbone comparison and most component ablations were conducted once per configuration and are therefore interpreted as descriptive point estimates.

Timestamp Conditioning does not improve upon the shared Swin-Unet baseline, suggesting that injecting a coarse acquisition-period embedding into one shared model is insufficient under the present setting. In contrast, PFT improves the mean IoU, Dice, Recall, BF1, and HD_95_, confirming the value of period-specific specialization, although its PE increases from 8.48% to 10.45%.

SPMES achieves the best mean result for all six metrics. Relative to Swin-Unet, it improves IoU, Dice, Recall, and BF1 by 1.55, 0.95, 2.56, and 3.94 percentage points, respectively, while reducing HD_95_ by 9.67 pixels and PE by 0.84 percentage points. Relative to PFT, it further improves IoU by 0.73 percentage points and BF1 by 1.64 percentage points and reduces HD_95_ and PE by 6.85 pixels and 2.82 percentage points, respectively. Because PFT and SPMES share the same GE-based initialization, expert number, and deterministic routing rule, these differences indicate benefits from retaining the GE prior branch and SGFM beyond period-wise fine-tuning alone.

The paired confidence intervals support the IoU, Dice, Recall, and BF1 improvements over Swin-Unet and the IoU, Dice, and BF1 improvements over PFT. The remaining intervals include zero and are therefore interpreted as favorable mean trends rather than statistically established differences. In particular, although the mean PE value favors SPMES over Swin-Unet, the corresponding 95% confidence interval includes zero; this change is therefore interpreted as a modest favorable mean trend rather than a statistically supported improvement. Given the three paired runs, the intervals provide focused uncertainty estimates rather than exhaustive statistical validation.

### 4.7. Effect of Input Resolution

The original monitoring images have a resolution of 2560×1440, whereas the default network input size is 320×320. Although the predicted masks are restored to the original image resolution before metric calculation, this operation only provides a common evaluation coordinate system and cannot recover fine spatial information that may already have been removed during input downsampling. To examine the influence of input resolution on thin water boundaries and delicate structures, Swin-Unet and the complete Swin-Unet-based SPMES were evaluated using input sizes of 320×320, 480×480, and 640×640.

All resolution-specific configurations were trained once using the same random seed, 3407. At each input resolution, the corresponding Swin-Unet baseline was trained first and subsequently used as the Global Expert for SPMES. The data partition, augmentation pipeline, patch size, window size, embedding dimension, stage depths, attention-head configuration, loss weights, optimization settings, training schedule, and acquisition-period partition were kept unchanged. Only the spatial input resolution and the resulting feature-map dimensions were varied. The default 320×320 setting used in the main experiments had been fixed before this additional analysis, and the test-set comparison was not used for input-resolution selection. Therefore, the experiment is interpreted as a controlled single-seed resolution analysis rather than a repeated-run statistical comparison.

As shown in Table 7, increasing the input resolution does not improve test-set boundary performance under the unchanged Swin-Unet architecture and training protocol. For Swin-Unet, BF1 decreases from 69.25% at 320×320 to 65.49% and 62.49% at 480×480 and 640×640, respectively, while HD_95_ increases from 85.04 to 94.89 and 103.43 pixels. The corresponding IoU decreases from 82.25% to 80.39% and 79.09%. For SPMES, BF1 decreases more moderately from 74.68% to 73.87% and 73.67%, while HD_95_ increases from 70.16 to 87.81 and 98.06 pixels. Its IoU decreases from 84.43% to 83.85% and 83.41%. These results show that simply increasing the number of input pixels does not guarantee improved thin-boundary recovery when the model architecture, local window size, optimization settings, and training schedule remain unchanged.

Across the three evaluated input resolutions, SPMES yields higher IoU and BF1 and lower HD_95_ than the corresponding Swin-Unet baseline. At 320×320, 480×480, and 640×640, the IoU differences are 2.18, 3.46, and 4.32 percentage points, respectively, while the BF1 differences are 5.43, 8.38, and 11.18 percentage points. The corresponding HD_95_ reductions are 14.88, 7.08, and 5.37 pixels.

When the input size increases from 320×320 to 640×640, Swin-Unet decreases by 3.16 percentage points in IoU and 6.76 percentage points in BF1, whereas SPMES decreases by 1.02 and 1.01 percentage points, respectively. Thus, within these single-run comparisons, SPMES shows smaller reductions in IoU and BF1 as the evaluated resolution increases. Nevertheless, HD_95_ deteriorates for both methods at higher resolutions, indicating that larger local boundary deviations cannot be eliminated solely through increased spatial sampling.

Increasing the input resolution raises the peak inference memory from 128.07 MiB to 302.49 MiB for Swin-Unet and from 198.42 MiB to 403.47 MiB for SPMES. The 640×640 configuration also produces the lowest inference speed for both methods. Under the current controlled architecture and training protocol, the default 320×320 setting therefore provides the best observed combination of region accuracy, boundary quality, and computational efficiency. This conclusion is restricted to the evaluated square-input configurations and does not imply that higher-resolution spatial information is intrinsically unhelpful.

### 4.8. Qualitative Analysis

**Figure 4:**
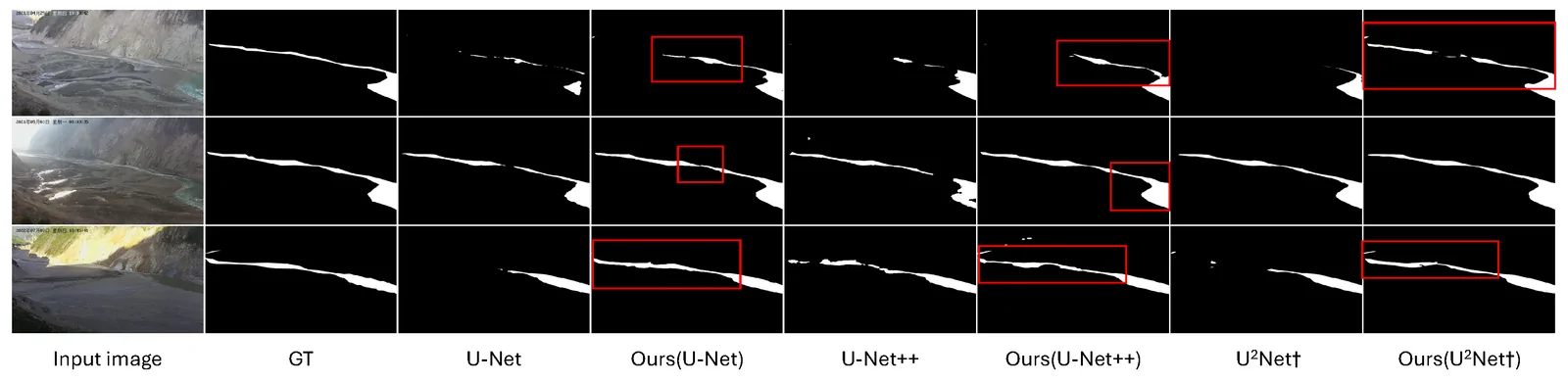
Qualitative comparison on three representative test samples. Columns from left to right: input image, Ground Truth (GT), U-Net, Ours (U-Net), U-Net++, Ours (U-Net++), U^2^-Net^†^, and Ours (U^2^-Net^†^). Red boxes highlight regions with notable differences among the compared methods. Here, Ours (·) denotes SPMES with the indicated backbone.

**Figure 5:**
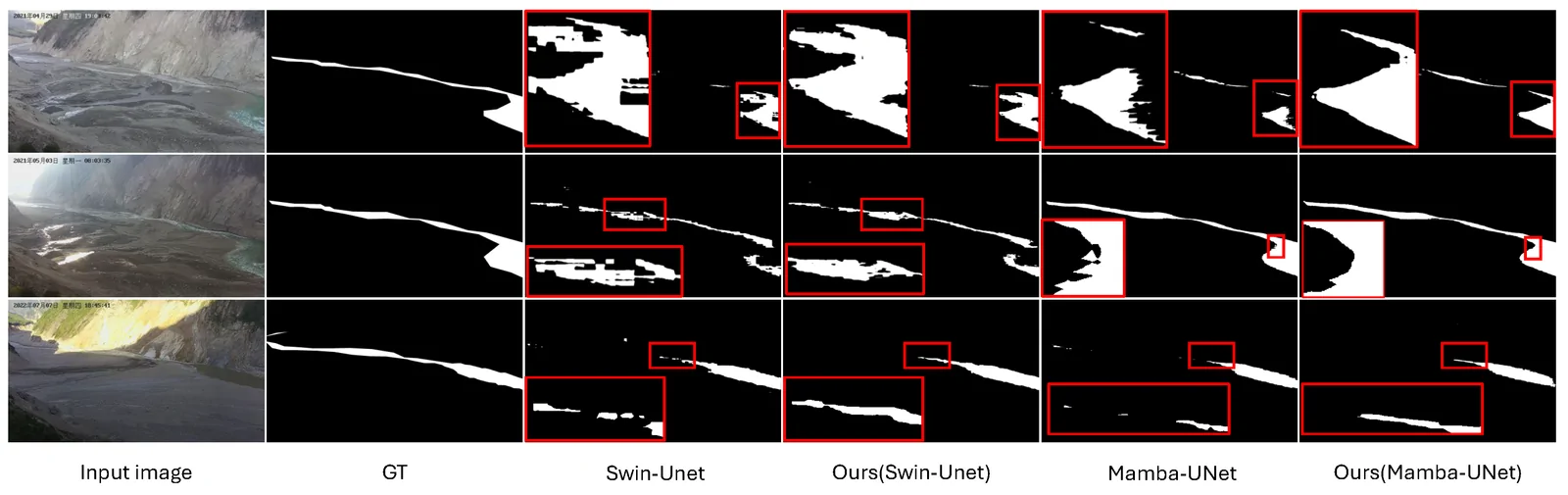
Qualitative comparison on three representative test samples. Columns from left to right: input image, Ground Truth (GT), Swin-Unet, Ours (Swin-Unet), Mamba-UNet, and Ours (Mamba-UNet). Red boxes and enlarged views indicate local differences. Here, Ours (·) denotes SPMES with the indicated backbone.

Figure 4 and Figure 5 present qualitative comparisons on three representative test samples. Figure 5 additionally provides enlarged local regions to highlight differences that are less apparent in the complete masks.

The examples suggest that SPMES can recover some locally omitted regions and reduce boundary discontinuities, although the magnitude of the visual difference varies across samples and backbones. The enlarged regions show that the improvements for Swin-Unet and Mamba-UNet are often localized rather than visually dominant over the entire mask. These observations are broadly consistent with the favorable BF1 and HD_95_ results, whereas the comparatively small PE changes should be interpreted cautiously.

### 4.9. Ablation Study

To isolate the contributions of period-specific specialization, semantic-prior guidance, and SGFM, five configurations are evaluated for each backbone: the original baseline; PSEs only; PSEs with SGFM but without the retained GE prior branch, where Value, Key, and Query are all derived from the input representation; Direct Semantic-Prior Concatenation (DSPC), which retains GE and the PSEs but replaces SGFM with channel concatenation followed by a 1×1 projection; and the complete SPMES, where Value and Key are derived from the input representation and Query is derived from the GE prior. The two intermediate configurations are parallel controls rather than successive stages, and the results are reported in Table 8.

Table 8 shows that PSE-only training improves IoU, Dice, Recall, and BF1 for all five backbones, confirming the value of period-specific specialization. However, HD_95_ and PE do not improve uniformly, indicating that better region overlap and local boundary agreement do not necessarily eliminate isolated or spatially displaced boundary errors. Adding SGFM without the retained GE prior branch also produces backbone-dependent trade-offs, showing that input-derived channel attention alone is insufficient to provide consistently favorable improvements.

DSPC likewise exhibits metric-dependent effects. For U-Net++, its IoU decreases slightly from 83.99% for PSE-only training to 83.80%, while HD_95_ and PE improve from 81.50 to 78.50 pixels and from 7.66% to 7.19%, respectively. Because DSPC is a parallel control rather than a successive stage of SPMES, this result represents a trade-off between region overlap and boundary geometry rather than an unexpected failure of a cumulative component. The result also suggests that direct prior concatenation can reduce large geometric deviations without consistently improving local overlap, potentially because imperfect prior predictions or feature-scale mismatch interfere with the period-specific representation.

The complete SPMES achieves the best point estimates across all six metrics for every evaluated backbone and outperforms both parallel controls. For Swin-Unet, relative to SGFM without the GE prior, IoU increases from 82.26% to 83.55%, BF1 from 70.15% to 72.66%, and HD_95_ decreases from 81.78 to 72.63 pixels. Relative to DSPC, the overlap improvements are modest, with IoU increasing from 83.48% to 83.55% and BF1 from 72.54% to 72.66%, but HD_95_ decreases markedly from 81.91 to 72.63 pixels. These results indicate that the GE prior and SGFM provide complementary benefits and that, for some backbones, their principal contribution lies in reducing large boundary deviations rather than substantially changing average region overlap. Since the component ablations were conducted once per configuration, these comparisons are interpreted descriptively rather than as statistically established differences.

### 4.10. Complexity Analysis

The computational cost of SPMES is evaluated using U-Net, Swin-Unet, and Mamba-UNet, representing CNN-, Transformer-, and state-space-model-based backbones, respectively. Table 9 reports loaded parameters, FLOPs, peak GPU memory, latency, and FPS at an input size of 320×320.

As shown in Table 9, SPMES approximately doubles the loaded parameters and FLOPs of each baseline because its inference path contains one frozen GE and one active PSE with the same backbone architecture. SGFM operates over the channel dimension and introduces only a comparatively small additional cost. DSPC and SPMES have identical loaded parameter counts and nearly identical FLOPs, while SPMES requires slightly more memory and latency because of its feature interaction and intermediate-feature storage.

Under these approximately parameter- and computation-matched conditions, SPMES outperforms DSPC across all six metrics for all five evaluated backbones. For Swin-Unet, the improvements over DSPC in region-overlap metrics are modest, whereas HD_95_ decreases from 81.91 to 72.63 pixels. This result indicates that the benefit of SPMES cannot be attributed solely to the increased model capacity and that SGFM is particularly useful for suppressing large boundary deviations. Nevertheless, the doubled backbone computation remains a cost, and SPMES should therefore be regarded as an accuracy–complexity trade-off rather than a universally parameter-efficient architecture.

The slowest evaluated configuration, Mamba-UNet with SPMES, requires 49.03 ms per image and achieves 20.40 FPS on the NVIDIA A30 GPU. This cost is acceptable for the current central-server pipeline, in which images are acquired and transmitted approximately once per hour, but it does not establish feasibility for resource-constrained edge devices, high-frequency emergency-warning systems, or multi-camera processing. Future work will investigate shared GE–PSE feature extraction, lightweight adapters or low-rank modules, a shallower or lower-resolution GE, knowledge distillation, mixed-precision inference, quantization, pruning, and operator fusion.

## 5. Discussion

The experiments suggest that the benefit of SPMES arises from combining period-specific specialization with global semantic-prior guidance. PSEs adapt the model to recurring appearance changes, whereas the frozen GE supplies a common structural prior and SGFM integrates it with the routed expert input. The ablation results show that neither input-derived channel attention nor direct prior concatenation achieves the same overall performance as the complete framework, indicating that both the source of the prior and the manner in which it is incorporated are important.

The effects are nevertheless metric- and backbone-dependent. PSE-only training generally improves overlap and local boundary agreement but may worsen HD_95_ or PE because isolated distant errors can remain. DSPC also exhibits trade-offs; for U-Net++, its IoU decreases slightly relative to PSE-only training while HD_95_ and PE improve. The complete SPMES obtains the best point estimates across all six metrics for every evaluated backbone, but some differences are modest. In particular, the Swin-Unet PE reduction is approximately 0.8 percentage points in both the main and repeated-run comparisons, and its paired confidence interval includes zero; it should therefore be interpreted as a favorable mean trend rather than strong evidence of a substantial perimeter improvement.

The dedicated repeated-run Swin-Unet experiments support several overlap and BF1 improvements, while some Recall, HD_95_, and PE intervals remain inconclusive with only three seeds. The five-backbone comparison, most component ablations, loss-weight analysis, and input-resolution analysis were conducted once per configuration and should be interpreted descriptively. The day-grouped split reduces the risk of leakage across subsets, while validation-based model selection, GE-based expert initialization, and the frozen GE prior help mitigate overfitting and avoid training each expert from random initialization. These safeguards do not eliminate possible scene-specific overfitting.

The current evidence is limited to one fixed camera and monitoring site and does not establish cross-camera, cross-site, or cross-sensor generalization. Camera displacement, changes in orientation, zoom, or field of view can spatially misalign the GE prior, while persistent morphology changes, severe occlusion, or sensor replacement can make it outdated or introduce a domain shift. In the current operational workflow, unusually large deviations from recent or historical segmentation patterns trigger manual inspection; confirmed camera or scene changes require recalibration, annotation updating, or retraining. Controlled geometric augmentation may improve tolerance to moderate displacement, but substantial deployment changes remain outside the current capability of SPMES.

The deterministic routing strategy further assumes that timestamps are available and that recurring variations can be approximated using predefined acquisition periods. The validation analysis supports T=4 within the examined range, but this setting is dataset-specific and does not represent exact climatic or hydrological boundaries. Future work should investigate data-driven selection of both the number and nonuniform boundaries of the periods, for example through temporal change-point detection or clustering of appearance representations using only the training and validation data.

SPMES also introduces a clear computational cost because one frozen GE and one active PSE are evaluated during inference. The parameter-matched DSPC comparison shows that the accuracy gain cannot be explained solely by the approximately doubled model capacity. Both SPMES and DSPC require approximately twice the computation of the corresponding backbone-only model. Compared with DSPC, SPMES has nearly identical FLOPs but slightly higher GPU-memory usage and inference latency. The measured cost is acceptable for the current central-server pipeline, where images are acquired and transmitted approximately once per hour, but it does not demonstrate suitability for edge devices, high-frequency warning, or multi-camera processing. Future work on deployment efficiency will investigate shared GE–PSE feature extraction, lightweight adapters or low-rank updates, a shallower or lower-resolution GE, distillation, quantization, pruning, mixed-precision inference, and operator fusion.

A controlled empirical comparison among channel, spatial, and hybrid attention mechanisms remains outside the scope of the present study. Although the channel-wise formulation of SGFM reduces the computational and memory cost relative to full pairwise spatial attention, this computational rationale does not establish that channel attention is generally more accurate or preferable. The current results therefore support the effectiveness of the adopted channel-attention-based SGFM within SPMES, but should not be interpreted as demonstrating its superiority over spatial or hybrid attention mechanisms. Future work will implement lightweight spatial and hybrid SGFM variants and compare their segmentation accuracy, parameter count, FLOPs, GPU-memory use, and inference latency under matched backbone, training, and evaluation settings.

Numerical comparison with domain-adaptation methods also remains outside the scope of the current experimental protocol. The present study considers fully supervised segmentation within one fixed-view monitoring scene, where all training images have pixel-level annotations and acquisition timestamps, without explicit source and target domains, unlabeled target-domain data, or cross-condition image correspondences. Consequently, the reported results should not be interpreted as evidence that SPMES outperforms domain-adaptation methods. Future work will establish explicit source–target evaluation settings across additional acquisition conditions, cameras, monitoring sites, or sensors, and compare representative domain-adaptation methods under harmonized data-access, supervision, backbone, and evaluation protocols.

## 6. Conclusions

This study presents SPMES, a semantic-prior-guided period-aware multi-expert framework for water-area segmentation under recurring environmental variations. The framework combines deterministic timestamp routing, period-specific experts, a frozen Global Expert, and SGFM-based prior interaction to coordinate stable scene structure with period-specific appearance representations.

Experiments with five representative backbones show that the complete SPMES obtains the best point estimates across all six metrics on the presented dataset. The ablations indicate that its gains cannot be explained solely by period-specific fine-tuning, input-derived channel attention, direct prior concatenation, or increased parameter capacity. A dedicated three-seed Swin-Unet analysis further supports the improvements in IoU, Dice, and BF1, although several Recall and geometric-boundary intervals remain inconclusive.

The present evidence is limited to one fixed camera and monitoring site, and evaluating both GE and one active PSE increases computational cost. Substantial viewpoint, sensor, or scene-layout changes may also invalidate the learned prior. The current study does not provide a controlled comparison with spatial or hybrid attention mechanisms, nor does it include numerical comparisons with domain-adaptation methods under an explicit source–target protocol. Future work will focus on cross-site validation, data-driven temporal partitioning, matched comparisons among channel, spatial, and hybrid attention mechanisms, domain-adaptation benchmarks under harmonized supervision and evaluation settings, lightweight GE–PSE sharing or adaptation, and deployment-oriented optimization for edge and higher-frequency monitoring scenarios.

## Figures and Tables

**Table 1:** Principal notation used in the formulation of SPMES.

Symbol	Dimension	Description
*C*	–	Number of channels in the input representation and the SGFM output.
$C_{G}$	–	Number of channels in the semantic-prior map. For binary segmentation, $C_{G}=1$.
$H^{\prime }\times W^{\prime }$	–	Spatial resolution of the input image and the semantic-prior map before SGFM resizing.
H×W	–	Unified spatial resolution used inside SGFM.
*X*	$C\times H^{\prime }\times W^{\prime }$	Input image tensor.
$X_{G}$	$C_{G}\times H^{\prime }\times W^{\prime }$	Soft semantic-prior map produced by GE.
$X^{\prime }$	C×H×W	Input tensor resized to the SGFM working resolution.
$X_{G}^{\prime }$	$C_{G}\times H\times W$	Semantic-prior map resized to the SGFM working resolution.
V,K,Q	C×(HW)	Value, Key, and Query representations used in the prior-guided channel attention.
*A*	C×C	Channel-wise semantic-guided attention matrix.
$F_{1}$	C×H×W	Attention-enhanced intermediate representation.
$F_{F}$	$(C+C_{G})\times H\times W$	Concatenated representation before channel projection.
$F_{O}$	C×H×W	Fused structure-enhanced representation produced by SGFM.
$M_{t}$	–	Period-specific expert assigned to the *t*-th acquisition period.
*Y*	–	Final segmentation prediction.

**Table 2:** Annotated-image distribution under the four-period setting.

Subset	Jan.–Mar.	Apr.–Jun.	Jul.–Sep.	Oct.–Dec.	Total
Training	255	257	256	257	1025
Validation	25	25	25	25	100
Test	58	59	59	59	235
Total	338	341	340	341	1360

**Note**: Calendar-day groups are assigned exclusively to one subset, independently of the acquisition-period labels used for expert routing.

**Table 3:** Validation-set sensitivity analysis of the BCE-to-Dice loss-weight ratio using the Swin-Unet-based Global Expert.

α:β	IoU (%) ↑	Recall (%) ↑	BF1 (%) ↑
1:5	79.16	83.03	71.33
1:3	80.55	82.67	70.22
1:1	80.44	83.06	69.57
3:1	81.08	82.25	70.41
5:1	81.24	**83.26**	**71.63**
7:1	**81.52**	82.58	70.18

**Note**: ↑ indicates that a higher value is better. Bold values indicate the best result for each metric.

**Table 4:** Comparison of segmentation performance across different backbone networks.

Backbone	Method	IoU ↑	Dice ↑	Recall ↑	BF1 ↑	HD_95_ (px) ↓	PE (%) ↓
U-Net [11]	Baseline	82.56	90.04	84.13	68.27	95.18	8.69
	+SPMES	**85.93**	**92.30**	**89.15**	**76.91**	**67.30**	**5.89**
U-Net++ [12]	Baseline	82.49	90.11	84.43	68.36	108.90	8.64
	+SPMES	**84.96**	**91.68**	**87.54**	**73.80**	**75.68**	**6.44**
U^2^-Net^†^ [13]	Baseline	83.59	90.93	85.19	69.77	84.05	8.64
	+SPMES	**85.72**	**92.20**	**88.20**	**75.36**	**67.97**	**6.09**
Swin-Unet [17]	Baseline	81.90	89.71	83.79	68.39	81.50	9.08
	+SPMES	**83.55**	**90.71**	**86.22**	**72.66**	**72.63**	**8.28**
Mamba-UNet [18]	Baseline	82.16	89.92	83.65	67.22	82.85	8.98
	+SPMES	**84.28**	**91.32**	**86.29**	**71.42**	**78.48**	**7.86**

**Note**: IoU, Dice, Recall, BF1, and PE are percentages; HD_95_ is measured in pixels at the original image resolution. Results are from one run per configuration unless otherwise stated; repeated three-seed results for selected Swin-Unet configurations are reported separately. The arrows ↑ and ↓ indicate that higher and lower values are better, respectively. Bold values indicate the better result within each backbone-specific comparison.

**Table 5:** Three-seed Swin-Unet comparison with temporal-conditioning and period-specific expert baselines (mean ± sample SD).

Method	IoU (%) ↑	Dice (%) ↑	Recall (%) ↑	BF1 (%) ↑	HD_95_ (px) ↓	PE (%) ↓
Swin-Unet	82.39 ± 0.21	90.00 ± 0.14	84.17 ± 0.24	69.54 ± 0.57	84.35 ± 3.55	8.48 ± 0.33
Timestamp Conditioning	81.60 ± 0.65	89.51 ± 0.41	83.29 ± 0.80	67.47 ± 1.65	90.63 ± 4.58	8.64 ± 0.20
Period-wise Fine-Tuning (PFT)	83.22 ± 0.48	90.52 ± 0.30	86.15 ± 0.29	71.84 ± 0.58	81.53 ± 0.55	10.45 ± 1.74
Swin-Unet + SPMES	**83.95** ± 0.42	**90.95** ± 0.26	**86.73** ± 0.62	**73.48** ± 1.07	**74.68** ± 4.05	**7.63** ± 0.18

**Note**: The arrows ↑ and ↓ indicate that higher and lower values are better, respectively. Bold values indicate the best mean result among the compared methods.

**Table 6:** Paired seed-level differences between SPMES and the comparison methods (mean and two-sided 95% paired-*t* CI).

Reference	ΔIoU	ΔDice	ΔRecall	ΔBF1	ΔHD_95_	ΔPE
Swin-Unet	1.55 [0.12,2.99]	0.95 [0.03,1.87]	2.56 [0.85,4.26]	3.94 [0.66,7.22]	9.67 [−6.21,25.56]	0.84 [−0.40,2.09]
PFT	0.73 [0.49,0.97]	0.43 [0.29,0.58]	0.58 [−0.31,1.48]	1.64 [0.07,3.21]	6.85 [−4.56,18.26]	2.82 [−1.08,6.72]

**Note**: Each cell reports the mean difference on the first line and the 95% CI on the second. Differences are SPMES minus the reference method for IoU, Dice, Recall, and BF1, and the reverse for HD_95_ and PE; positive values therefore favor SPMES. Intervals excluding zero indicate metric-level statistical support.

**Table 7:** Resolution analysis of Swin-Unet and SPMES using seed 3407; predictions were restored to 2560×1440 before evaluation.

Input Size	Method	IoU (%) ↑	BF1 (%) ↑	HD_95_ (px) ↓	Peak Memory (MiB) ↓	FPS ↑
320×320	Swin-Unet	82.25	69.25	85.04	128.07	76.44
	SPMES	**84.43**	**74.68**	**70.16**	198.42	36.46
480×480	Swin-Unet	80.39	65.49	94.89	199.57	74.91
	SPMES	**83.85**	**73.87**	**87.81**	288.33	35.88
640×640	Swin-Unet	79.09	62.49	103.43	302.49	59.77
	SPMES	**83.41**	**73.67**	**98.06**	403.47	28.35

**Note**: At each resolution, the trained Swin-Unet baseline also serves as the GE for SPMES. Results are controlled single-seed comparisons rather than repeated-run estimates. The arrows ↑ and ↓ indicate that higher and lower values are better, respectively. Bold values indicate the better segmentation result within each input-resolution pair.

**Table 8:** Ablation study of PSEs, the GE prior branch, and SGFM across different segmentation backbones.

Backbone	PSEs	GE Prior	SGFM	IoU ↑	Dice ↑	Recall ↑	BF1 ↑	HD_95_ (px) ↓	PE (%) ↓
U-Net [11]	×	×	×	82.56	90.04	84.13	68.27	95.18	8.69
	✓	×	×	84.18	91.03	86.60	73.49	75.85	7.25
	✓	×	✓	84.14	91.20	86.68	74.02	71.50	6.35
	✓	✓	×	84.83	91.65	87.15	74.09	70.77	7.10
	✓	✓	✓	**85.93**	**92.30**	**89.15**	**76.91**	**67.30**	**5.89**
U-Net++ [12]	×	×	×	82.49	90.11	84.43	68.36	108.90	8.64
	✓	×	×	83.99	91.03	86.19	72.12	81.50	7.66
	✓	×	✓	83.02	90.37	85.17	70.49	82.64	6.65
	✓	✓	×	83.80	91.01	85.69	70.62	78.50	7.19
	✓	✓	✓	**84.96**	**91.68**	**87.54**	**73.80**	**75.68**	**6.44**
U^2^-Net^†^ [13]	×	×	×	83.59	90.93	85.19	69.77	84.05	8.64
	✓	×	×	85.02	91.74	87.56	74.52	87.33	7.14
	✓	×	✓	84.13	91.21	86.24	71.75	79.97	6.25
	✓	✓	×	85.10	91.84	87.43	73.60	68.75	6.93
	✓	✓	✓	**85.72**	**92.20**	**88.20**	**75.36**	**67.97**	**6.09**
Swin-Unet [17]	×	×	×	81.90	89.71	83.79	68.39	81.50	9.08
	✓	×	×	82.97	90.29	85.28	71.51	89.29	9.09
	✓	×	✓	82.26	89.80	84.76	70.15	81.78	10.15
	✓	✓	×	83.48	90.66	86.20	72.54	81.91	8.38
	✓	✓	✓	**83.55**	**90.71**	**86.22**	**72.66**	**72.63**	**8.28**
Mamba-UNet [18]	×	×	×	82.16	89.92	83.65	67.22	82.85	8.98
	✓	×	×	84.09	91.01	86.18	70.87	79.41	8.56
	✓	×	✓	82.57	90.18	84.52	69.00	81.34	8.02
	✓	✓	×	84.18	91.27	86.03	70.73	79.14	8.53
	✓	✓	✓	**84.28**	**91.32**	**86.29**	**71.42**	**78.48**	**7.86**

**Note**: ✓/× denote enabled/disabled components, and “GE Prior” indicates retention of the frozen GE branch. All PSEs are initialized from the pretrained GE. IoU, Dice, Recall, BF1, and PE are percentages; HD_95_ is measured in pixels at the original image resolution. Results are single-run test-set averages. DSPC and SPMES have identical loaded backbone capacity and differ primarily in the prior-fusion mechanism. The arrows ↑ and ↓ indicate that higher and lower values are better, respectively. Bold values indicate the best result among the evaluated configurations for each backbone.

**Table 9:** Model complexity and parameter-matched comparison (input size = 320×320).

Method	Loaded Params (M)	FLOPs (G)	Peak Memory (MiB)	Latency (ms)	FPS
U-Net	31.04	75.57	445.97	7.91	126.34
U-Net + DSPC	62.09	151.16	571.75	15.96	62.67
U-Net + SPMES	62.09	151.18	585.01	16.33	61.22
Swin-Unet	15.24	6.65	128.07	13.08	76.44
Swin-Unet + DSPC	30.48	13.31	192.87	26.81	37.29
Swin-Unet + SPMES	30.48	13.34	198.42	27.43	36.46
Mamba-UNet	28.00	12.21	276.39	24.28	41.19
Mamba-UNet + DSPC	56.01	24.42	419.27	47.27	21.16
Mamba-UNet + SPMES	56.01	24.45	424.15	49.03	20.40

**Note**: DSPC and SPMES load one frozen GE and one active PSE; other PSE checkpoints remain in storage. Parameters and FLOPs were estimated with THOP. Peak memory and batch-size-one FP32 speed were measured on one NVIDIA A30 under torch.inference_mode() after 50 warm-up and 200 timed passes. Latency excludes data loading, preprocessing, host-to-device transfer, and satellite communication.

## Data Availability

The data presented in this study are available on request from the corresponding author due to restrictions related to the data-management policy of the long-term monitoring project. The raw monitoring images were collected from a fixed-position visual monitoring system deployed at an ice-avalanche-induced river blockage site and may contain sensitive information about the monitoring site and fixed surveillance facilities. Data may be made available upon reasonable request and with permission from the relevant project administrator.

## Abbreviations

The following abbreviations are used in this manuscript:

SPMES	Semantic Prior-Guided Period-Aware Multi-Expert Segmentation
DSPC	Direct Semantic-Prior Concatenation
PFT	Period-wise Fine-Tuning
GE	Global Expert
SGFM	Semantic-Guided Fusion Module
PSE	Period-specific Expert
IoU	Intersection over Union
BF1	Boundary F1-score
HD_95_	95% Hausdorff Distance
PE	Perimeter Error
GT	Ground Truth
FLOPs	Floating-Point Operations
FPS	Frames per Second
GPU	Graphics Processing Unit
FP32	32-bit Floating-Point Precision
